# Early clinical outcomes of restor3D custom three dimensional-printed glenoid components in complex shoulder arthroplasty

**DOI:** 10.1016/j.jsea.2026.100042

**Published:** 2026-06-02

**Authors:** Ben Wesorick, Tyler Hasbrook, Carter Jerman, Aaron Baessler, Brad Crackel, Molly Moor, Scott Stephens, Brian Badman

**Affiliations:** a Weill Cornell Medical College, New York, NY, USA; b Indiana University, Bloomington, IN, USA; c Central Indiana Orthopedics, Fishers, IN, USA; d OrthoNeuro, New Albany, OH, USA

**Keywords:** Reverse shoulder arthroplasty, Glenoid bone loss, Patient-specific implants, Custom glenoid implant, 3D printing / Additive manufacturing, Glenoid fixation, Implant failure

## Abstract

**Background:**

Severe glenoid bone loss in primary or revision reverse total shoulder arthroplasty presents significant surgical challenges. Patient-specific 3D-printed glenoid implants offer customized fixation strategies for complex anatomical deficits, including modified peg designs, additional screws, and proprietary ingrowth surfaces. Despite promising early results, concerns regarding cost, surgical accuracy, and long-term stability remain.

**Methods:**

This retrospective study evaluated clinical and radiologic outcomes in patients with severe glenoid bone loss who underwent primary or revision reverse total shoulder arthroplasty with a custom three dimensional-printed glenoid implant (restor3d) by a single surgeon (December 2022–July 2024). Demographics, surgical indications, implant specifications, patient-reported outcomes, imaging, and complications were collected. Patients with and without complications were compared using Mann-Whitney *U* and Fisher's exact tests.

**Results:**

Eighteen patients (11 women, 7 men; median age 69 years [interquartile range, 67–77]; mean follow-up 18 ± 7 months) were included. Five patients underwent primary surgery and 13 underwent revision procedures, including 7 (39%) staged revisions. Complications occurred in 27.8% of patients: broken inferior or posterior screws (n = 1) and clinical implant failures, including broken central posts or fibrous ingrowth along the post (n = 4). The 4 patients with implant failure required explantation and revision at a mean of 21.7 months. Patients with complications reported greater post-operative visual analog scale pain (*P* = .051) and worse American Shoulder and Elbow Surgeons scores (*P* = .075). A greater quantity and longer length of ≥4.5 mm screws were each significantly associated with fewer complications (*P* = .041 and *P* = .035, respectively). Total screw engagement volume in bone was significantly greater in constructs without complications versus those with complications (4315 mm^3^ vs. 2,824 mm^3^, *P* = .018).

**Discussion:**

Custom 3D-printed glenoid implants show potential for addressing severe glenoid bone loss with patient-specific designs that enhance initial stability. Total screw engagement volume was significantly greater in constructs without complications (*P* = .018). The data would suggest that larger screws are associated with lower complication rates and improved implant stability compared to 4.0 mm screws; however, this may reflect increased residual bone stock rather than a causal relationship. The 27.8% complication rate in this cohort warrants further monitoring. Future studies with larger cohorts are needed to validate these findings and optimize fixation strategies.

Severe glenoid bone loss presents a demanding challenge in shoulder arthroplasty, particularly in the context of revision surgery. Conventional techniques, including eccentric reaming, bone grafting, and structural reconstruction with iliac crest autografts or allografts, have significant limitations in achieving stable fixation and restoring anatomical joint orientation, especially when host bone stock is critically compromised.^7^ Outcomes remain variable, with complications such as graft resorption, implant loosening, and donor-site morbidity presenting major obstacles to consistent success.^4^^,^^5^^,^^10^ In case series involving bone grafting for massive glenoid defects, graft resorption has been reported in up to 25% of cases by 2 years, consistent with radiographic failure.^6^ Additionally, glenoid allografting demonstrates inferior 5-year survivorship (78.4%) compared to autografting (95%) in the setting of primary reverse total shoulder arthroplasty (rTSA).^13^ As the prevalence of complex primary and revision rTSA continues to grow, the demand for more reliable and durable solutions to manage massive glenoid bone loss has become increasingly urgent.^10^

Custom glenoid implants represent a promising evolution in shoulder arthroplasty. By leveraging patient-specific computed tomography (CT) data and additive manufacturing, these implants can be designed to match individual bone loss patterns with high precision. They allow for optimized screw trajectory planning, enhanced mechanical stability, and porous surface coatings that promote osseointegration. These features are particularly advantageous in cases of severe glenoid erosion, prior failed grafting, or inadequate bone stock.

Recent literature supports the feasibility of this approach. In a review of patients treated with patient-specific glenoid implants across early and recent case series, the overall complication rate was 12%, with a glenoid loosening rate of 3% and a revision rate of 7% with a mean follow-up of 32 months.^14^ Modern systems like the Lima ProMade and Zimmer VRS have demonstrated 0% revision rate in some series and 0–5% glenoid failure at short-term follow-up, despite severe deformity patterns such as Walch C, Antuna severe combined, or Seebauer E4 morphology.^10^ These results reflect the potential short-term benefits of custom implants, particularly in achieving immediate stable fixation in challenging glenoid anatomy.

Despite early enthusiasm, the widespread adoption of this technology has been tempered by questions about cost-effectiveness, surgical reproducibility, implant longevity, and complication profiles, many of which remain inadequately addressed in existing literature.^3^ This study aims to contribute to the existing body of evidence by presenting early clinical and radiographic outcomes from a single surgeon who has undergone primary or revision rTSA using custom three dimensional (3D)-printed glenoid implants. By examining complication rates and implant failures, along with patient-reported outcome measures (PROMs), we aim to understand the benefits and initial limitations of customized implants for patients with significant glenoid bone loss. We particularly focus on evaluating the relationship between fixation strategy (eg, screw type and design) and implant success, with the goal of refining indications and improving technical guidelines for future use of this emerging technology in complex shoulder reconstruction.

## Methods

This retrospective cohort study evaluated a consecutive series of patients with severe glenoid bone loss who underwent primary or revision rTSA using a custom 3D-printed glenoid implant (restor3d, Durham, NC). All procedures were performed by a single fellowship-trained shoulder arthroplasty surgeon between December 2022 and July 2024 at a single academic institution. The decision to utilize a custom implant was based on pre-operative CT evaluation demonstrating severe glenoid deformity or insufficient bone stock that was not amenable to fixation with a standard glenoid baseplate. Indications for surgery, prior implant status, and glenoid deformity parameters (glenoid version and inclination) are reported in Table I to characterize the degree and type of glenoid pathology treated in this cohort. Institutional review board approval was obtained prior to data collection. Patients were included if they underwent rTSA with a custom glenoid implant designed to address complex bone loss, either in the primary or revision setting. All patients received pre-operative planning with CT-based modeling and had a minimum follow-up of 12 months. Demographic data, surgical indications, comorbidities, and implant design specifications were collected. Clinical outcomes included pre-operative and post-operative PROMs, such as American Shoulder and Elbow Surgeons and visual analog scale (VAS) pain scores, and active range of motion. Post-operative complications were documented, including infection, instability, hardware failure, and the need for revision surgery.

**Table I:** Patient indications and surgical parameters.

Age, years	Sex	Follow-up, months	Prior implant status	Indication for surgery	Inclination	Retroversion
76	F	37	Primary	Rotator cuff arthropathy	5° inferior tilt	0° retroversion
67	M	38	Antibiotic spacer	Failed aTSA	23° superior tilt	5° retroversion
74	M	35	Antibiotic spacer	Failed aTSA	17° superior tilt	22° retroversion
62	F	34	Antibiotic spacer	Failed rTSA	7° inferior tilt	7° retroversion
80	F	36	Primary	Rotator cuff arthropathy	38° superior tilt	6° retroversion
70	M	33	Antibiotic spacer	Failed aTSA	42° superior tilt	2° retroversion
67	M	33	Primary	Severe glenoid deformity	13° inferior tilt	34° anteversion
81	F	33	Primary	Severe glenoid deformity	37° superior tilt	20° retroversion
66	M	33	aTSA	Failed aTSA w/loosening	9° superior tilt	21° retroversion
71	F	32	Antibiotic spacer	Failed aTSA	22° superior tilt	15° retroversion
67	F	31	Hemiarthroplasty	Intraoperative glenoid fracture	12° superior tilt	21° retroversion
67	M	29	Antibiotic spacer	Failed rTSA for infection	20° superior tilt	10° retroversion
78	F	26	aTSA	Failed aTSA w/loosening	12° superior tilt	21° retroversion
58	M	26	Antibiotic spacer	Failed rTSA w/loosening	1° inferior tilt	8° retroversion
78	F	23	aTSA	Failed aTSA w/loosening	6° superior tilt	24° anteversion
68	M	21	Hemiarthroplasty	Failed hemiarthoplasty	8° inferior tilt	31° anterversion
67	F	15	aTSA	Failed aTSA w/loosening	17° superior tilt	0° retroversion
70	F	18	rTSA	Failed rTSA w/loosening	18° superior tilt	4° retroversion

*aTSA*, anatomic total shoulder arthroplasty; *rTSA*, reverse total shoulder arthroplasty.

To evaluate fixation characteristics, pre-operative implant design plans were analyzed using Materialise 3-matic software (Leuven, Belgium). For each patient-specific implant, screw diameters, planned screw lengths, and the in-bone screw lengths, and central post lengths were extracted from the finalized design files. Each screw was modeled as a uniform cylinder, and the total screw engagement volume in bone was approximated using the formula π*r*^*2*^*h*, where *r* is the inner screw radius, and *h* is the planned in-bone length.^1^^,^^15^ The sum of these values provided a total screw volume in bone for each construct, allowing comparison between patients who experienced complications and those who did not. Planned screw lengths were obtained from finalized pre-operative CT-based implant design files. Actual intraoperative screw lengths were extracted from operative records when available. For each screw, the absolute deviation between planned and actual length was calculated. Summary metrics included median absolute deviation and the proportion of screws placed within ±2 mm and ±5 mm of the planned length. Descriptive comparisons were performed between screws from patients with and without post-operative complications. This analysis was intended to assess execution fidelity of pre-operative planning rather than serve as a predictive model for complications.

Since the data were nonparametric, comparisons between patients with and without complications were performed using Fisher's exact test for categorical variables, and Mann-Whitney U tests for continuous variables. All tests were 2-tailed, and statistical significance was defined as *P* < .05. Data were analyzed using SPSS Version 29 (IBM Corp., Armonk, NY).

## Results

**Figure 1:**
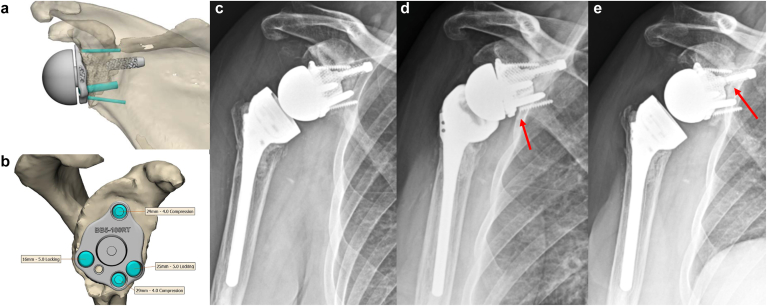
(**a**) Pre-operative plan demonstrating glenosphere placement and final implant construct. (**b**) Pre-operative plan showing screw configuration. (**c**) Immediate post-operative anteroposterior (AP) radiograph. (**d**) 12-month post-operative AP radiograph demonstrating a broken screw (red *arrow*). (**e**) 18-month post-operative AP radiograph demonstrating a nondisplaced broken post (red *arrow*).

**Figure 2:**
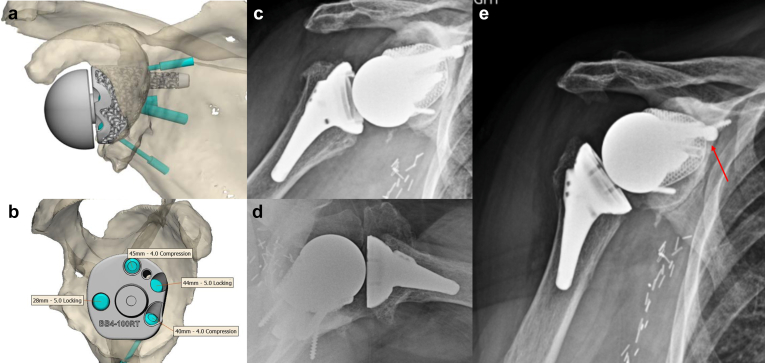
(**a**) Pre-operative plan demonstrating glenosphere placement and final implant construct. (**b**) Pre-operative plan showing screw configuration. (**c**) Immediate post-operative anteroposterior (AP) radiograph. (**d**) Immediate post-operative axillary radiograph (alternate view). (**e**) 12-month post-operative AP radiograph demonstrating a broken post (red *arrow*).

Eighteen patients (11 women and 7 men) with a median age of 69 years (interquartile range [IQR], 67–77) and a mean follow-up time of 18 ± 7 months were included. Five (28%) patients underwent primary surgery, and 13 (72%) underwent revision procedures, with 7 of these (39%) performed as staged revisions. Fourteen patients received a post-operative CT scan at an average of 6.8 months (range: 3–13 months). Overall, complications occurred in 27.8% (5/18) of patients. These included broken inferior or posterior screws (n = 1) and clinical implant failures (n = 4). One patient was noted to have a baseplate failure and a subsequent failed post, as shown in Figure 1. The implant failures necessitated explantation and revision at a mean of 21.7 months with an overall revision rate of 22.2%. Implant failures included 2 patients with broken central posts and 2 patients with noted fibrous ingrowth along the post. Complications were noted at a mean of 15 months from index surgery, while patients without complications had an average follow-up of 22.6 months. Table II shows the different cohorts and their complications. Figures 1 and 2 show radiographs of patients who had complications at different time points.

**Table II:** Patient characteristics across the entire cohort.

Variable	All patients (N = 18) median (IQR) or mean ± SD or n (%)
Age (yr)	69 (67-77)
Female sex	11 (61.1)
Operative side	
Right	13 (72.2)
Left	5 (27.8)
Complications (total)	5 (27.8)
Broken inferior or posterior screw	1 (5.6)
Implant failure	4 (22.2)
Broken central post	2 (11.1)
Fibrous ingrowth on post	2 (11.1)
Length of follow-up (mo)	18 ± 7

*IQR*, interquartile range; *SD*, standard deviation.

Although not statistically significant, patients with complications reported greater post-operative VAS pain (*P* = .051) than patients without complications, as shown in Table III. Furthermore, complications were observed exclusively in female patients (*P* = .101) but this did not reach statistical significance. Additionally, there was a trend toward complications in patients with comorbidities, including diabetes (*P* = .065), rheumatoid arthritis (*P* = .172), and osteoporosis (*P* = .172), however these were not statistically significant. Age and both pre-operative and post-operative PROMs and ranges of motion were comparable between patients with and without complications.

**Table III:** Outcome scores for patients by complication status.

Variable	Complications (N = 5) median (IQR) or n (%)	No complications (N = 13) median (IQR) or n (%)	*P* value
Age (yr)	71 (65-81)	68 (67-75)	.483
Female sex	5 (100)	6 (46.2)	.101
Operative side			1.00
Right	4 (80.0)	9 (69.2)	
Left	1 (20.0)	4 (30.8)	
Primary surgery	2 (40.0)	3 (23.1)	.583
Positive bacterial culture	1 (20.0)	4 (30.8)	1.00
Pre-operative ASES	27 (16-41)	35 (23-45)	.387
Pre-operative SST	17 (0-32)	29 (12-45)	.274
Pre-operative VAS pain	6 (4-9)	7 (5-8)	.782
Pre-operative forward flexion	80 (65-95)	85 (60-125)	.651
Pre-operative abduction	75 (50-85)	80 (55-133)	.428
Pre-operative external rotation	20 (5-30)	35 (8-50)	.297
Pre-operative internal rotation > buttock	0 (0)	5 (38.5)	.249
Post-operative ASES	41 (29-75)	74 (59-83)	.075
Post-operative SST	66 (31-75)	72 (57-92)	.426
Post-operative VAS pain	5 (2-7)	1 (0-2)	.051
Post-operative forward flexion	120 (80-145)	130 (105-150)	.575
Post-operative abduction	90 (70-140)	110 (80-136)	.752
Post-operative external rotation	30 (25-63)	55 (15-65)	.941
Post-operative internal rotation > buttock	4 (80.0)	9 (69.2)	1.00
Comorbidities			
Hypertension	3 (60.0)	11 (84.6)	.533
Diabetes	2 (40.0)	0 (0)	.065
Cancer	0 (0)	2 (15.4)	.575
Smoker (current or former)	1 (20.0)	1 (7.7)	.490
Rheumatoid arthritis	2 (40.0)	1 (7.7)	.172
Osteoporosis	2 (40.0)	1 (7.7)	.172

*IQR*, interquartile range; *ASES*, American Shoulder and Elbow Surgeons; *SST*, Simple Shoulder Test; *VAS*, visual analog scale.

**Figure 3:**
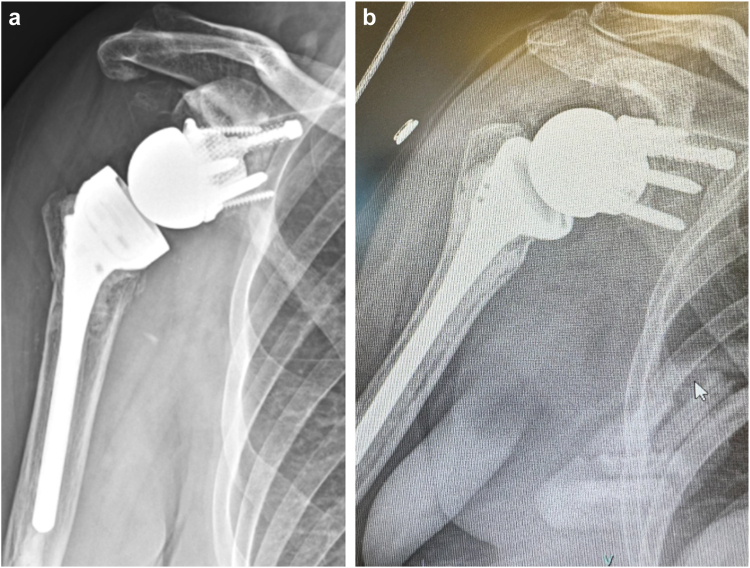
(**a**) Immediate post-operative radiograph demonstrating a combination of 4.0 mm and 5.0 mm screws. (**b**) Immediate post-operative radiograph demonstrating fixation with all 5.0 mm locking screws.

Implant fixation analysis, shown in Table IV, revealed that patients who were treated with both a greater quantity and longer length of ≥4.5 mm screw experienced significantly fewer post-operative complications (*P* = .041 and *P* = .035, respectively). Implants without complications had a median of 3 ≥4.5 mm screws compared with 2 screws in the complication group, and the total length of ≥4.5 mm screws was 90 mm versus 59 mm, respectively. Furthermore, all implants which ultimately failed utilized at least one 4.0 mm screw (*P* = .101), and there was no statistically significant difference in either the total quantity or length of 4.0 mm screws used between patients with and without complications (*P* = .137 and *P* = .240). Figure 3 compares an implant with 4.0 mm screws to an implant designed with 5.0 mm screws. When total screw engagement volume in bone was evaluated, constructs in the non-complication group demonstrated significantly greater total screw volume in bone compared with those in the complication group (4315 mm^3^ vs. 2,824 mm^3^, *P* = .018). There was no significant difference in central post diameter or length between groups.

**Table IV:** Implant fixation characteristics by complication status.

Variable	Complications (N = 5) median (IQR) or n (%)	No complications (N = 13) median (IQR) or n (%)	*P* value
Glenosphere size >40 mm	0 (0)	5 (38.5)	.249
Post diameter (mm)			1.00
6.0	0 (0)	1 (7.7)	
7.3	5 (100)	12 (92.3)	
Post length (mm)	25 (19-34)	26 (19.5-30.5)	.868
Total number of screws used	4 (3-4)	4 (3.5-4)	.634
4.0 mm screws used (yes)	5 (100)	6 (46)	.101
Number of 4.0 mm screws used	2 (1.5-2)	0 (0-2)	.137
Total length (mm) of 4.0 mm screws used	59 (42-80)	82 (55-88)	.240
Number of ≥4.5 mm screws used	2 (2-2)	3 (2-4)	.041
Total length (mm) of ≥4.5 mm screws used	59 (35-68)	90 (65-137)	.035
Total screw lengths (mm)	100 (86-148)	133 (111-161)	.246
Total screw length in bone (mm)	81 (58-81)	89 (70.8-114.0)	.289
Total screw engagement volume in bone (mm^3^)	2,824 (2,307-3,356)	4315 (2,810- 5,569)	.018

*IQR*, interquartile range.

Planned versus actual screw length analysis demonstrated high concordance. Across 37 screws for which intraoperative length data were available, the median absolute difference between planned and actual screw length was 1.0 mm, with 68% of screws placed within ±2 mm and 89% within ±5 mm of the planned length. Screws from patients who experienced complications demonstrated greater variability (median absolute difference 3.0 mm, IQR, 1.1–4.5 mm) compared with screws from patients without complications (median 1.0 mm, IQR, 0–2.75 mm); however, this difference was not statistically significant, potentially due to the small sample size (*P* = .094).

## Discussion

This study presents early clinical and radiographic outcomes of custom 3D-printed glenoid implants in patients with severe glenoid bone loss undergoing primary or revision rTSA. With a mean follow-up of 18 months, the overall complication rate was 27.8%, including a 22.2% implant failure rate necessitating revision surgery, which is higher than what has previously been reported for custom glenoid implants. Despite these complications, patients without severe adverse events demonstrated meaningful improvements in shoulder function and pain, as reflected by a median post-operative American Shoulder and Elbow Surgeons score of 74 and VAS pain score of 1. These results are consistent with those reported in previous studies of 3D-printed glenoid components, which have shown promising short-term outcomes in both primary and revision settings for severe bone loss.^3^^,^^6^^,^^10^^,^^11^^,^^14^

The functional improvements observed may be largely attributed to the correction to ideal anatomy that occurs with custom implants. Designed to match the patient's native glenoid morphology, these devices allow for optimized screw trajectory and maximal host bone contact, facilitating immediate mechanical stability while allowing correction of glenoid deformity. Porous surface coatings further enhance osseointegration and may reduce micromotion over time.^4^ This biomechanical advantage has been confirmed in both in vitro and in vivo models, supporting the rationale for using additive-manufactured designs in high-risk glenoids.^11^^,^^12^ While our patient cohort experienced early pain relief and restoration of function, longer-term follow-up will be necessary to determine implant survival and biological integration rates over time.

Implant failures in this cohort included 2 cases of central postfracture and fibrous ingrowth at the implant–bone interface. Central post breakage likely reflects inadequate osseointegration or insufficient biologic fixation, resulting in increased mechanical stress and eventual structural failure. Similarly, cases of fibrous ingrowth suggest failure of bony integration and compromised implant stability. These findings indicate that implant failure in this cohort was primarily related to inadequate biologic fixation rather than purely mechanical factors and highlight the importance of achieving reliable osseointegration, particularly in patients with severely compromised glenoid bone stock. This could suggest insufficient bone ingrowth material on the implant or insufficient primary fixation to promote osseointegration.^8^

A key finding of this study was the association between fixation characteristics and clinical outcomes. Total screw engagement volume in bone was significantly greater in constructs without complications compared to those with complications (median 4315 mm^3^ vs. 2,824 mm^3^, *P* = .018). Because engagement volume increases with both screw radius and in-bone length, this finding supports the importance of maximizing screw diameter and depth of purchase when designing fixation strategies for severe glenoid bone loss. This interpretation aligns with prior biomechanical literature demonstrating that increasing screw length significantly reduces baseplate micromotion. In one study, increasing screw length from 18 mm to 30 mm and 46 mm progressively decreased micromotion at the bone–implant interface, supporting the notion that greater screw engagement can enhance primary stability and reduce early failure risk.^9^^,^^12^ In our cohort, the significantly decreased complication rate among patients with larger and longer screws could reflect a biomechanically meaningful threshold for stable fixation. However, fixation characteristics in this study were primarily derived from pre-operative CT-based implant design files rather than direct post-operative 3-dimensional assessment of final implant position. Although planned versus actual screw length analysis demonstrated concordance in a subset of cases, intraoperative execution may differ from the pre-operative plan, and these findings should therefore be interpreted with caution.

Additionally, the trend toward higher complication rates in implants using predominantly smaller screws echoes prior clinical findings. The use of greater quantities and lengths of ≥4.5 mm screws for implant fixation was significantly associated with fewer post-operative complications. Studies have shown that baseplate fixation using 5.0 mm screws, particularly when locking and engaging cortical bone, yields superior initial stability and reduced rates of aseptic loosening compared to smaller 3.5 mm screws. Use of exclusively 3.5 mm non-locking screws has been identified as an independent risk factor for aseptic baseplate loosening, with an odds ratio as high as 10.6, while 5.0 mm locking screws significantly reduce this risk.^2^^,^^9^ Longer screws that achieve cortical purchase have similarly been associated with improved construct stability and reduced micromotion, both of which are critical for osseointegration and long-term implant survival. Together, these results highlight the potential importance of maximizing screw diameter, length, and fixation in bone when designing custom glenoid implants for patients with compromised bone stock.

In contrast to structural bone grafting techniques, which have historically been used to address massive glenoid defects, custom 3D-printed implants offer several theoretical advantages. Graft-based reconstructions carry well-documented risks of resorption, nonunion, hardware loosening, and donor site morbidity. In one large study of structural grafting in rTSA, early radiographic failure occurred in 25% of cases due to graft resorption and loss of fixation.^6^ More recently, Tashjian et al reported minimum 5-year outcomes of structural bone grafting for severe glenoid defects, demonstrating an overall baseplate failure rate of approximately 22% and 5-year survivorship of 78%.^4^ Failure rates were higher in revision cases and when allograft was utilized. These findings demonstrate that graft-based reconstruction remains a viable long-term option in selected patients but is not without meaningful risk of mechanical failure. While custom implants eliminate the need for structural graft incorporation and allow immediate rigid fixation to host bone, long-term success still depends on reliable osseointegration at the implant–bone interface. The failure rate in our cohort underscores that biologic fixation and bone quality remain critical determinants of success regardless of reconstruction strategy. Direct comparative studies are needed to determine whether custom implants provide superior long-term durability compared to modern grafting techniques.

This study has several important limitations. First, the cohort was heterogeneous, including both primary and revision reverse shoulder arthroplasty cases across a range of ages, indications, and degrees of bone loss. Although this reflects real-world clinical practice in managing severe glenoid deformity, the variability in surgical indication and prior implant status may introduce confounding factors that influence outcomes. Second, the retrospective design and small sample size limit statistical power and preclude multivariable adjustment, and associations between fixation characteristics and complications should therefore be interpreted as exploratory rather than causal. Third, fixation and implant design analyses were primarily derived from pre-operative CT-based implant planning rather than a comprehensive post-operative 3-dimensional assessment of the final implant position. Although patient-specific instrumentation is intended to closely replicate the planned construct, intraoperative execution may differ. Planned versus actual screw length analysis demonstrated general concordance in a subset of screws; however, complete three-dimensional post-operative modeling was not performed, and subtle differences in screw trajectory, cortical engagement, and final fixation configuration could not be fully quantified. Post-operative CT imaging was available only in a subset of patients and was not obtained in all patients nor at a consistent time point, limiting more robust radiographic analysis of implant positioning and osseointegration. Additionally, implants included in this study were from a single manufacturer (restor3d), which limits generalizability to other custom implant designs. Finally, follow-up duration was relatively short, restricting assessment of long-term implant survivorship, biologic integration, and late failure modes.

This study contributes to the growing evidence of the use of patient-specific 3D-printed implants in shoulder arthroplasty for severe glenoid bone loss. While our findings echo those of other studies showing early success, they also raise important concerns about implant survivability and complication risk.^3^^,^^10^^,^^11^^,^^14^ Surgeons should approach fixation planning with caution in patients with compromised bone quality, particularly postmenopausal women or those with inflammatory or metabolic bone disease. Emphasis should be placed on maximizing screw length, diameter, and total fixation to enhance initial mechanical stability in these challenging cases. Future investigations with larger cohorts and long-term follow-up are needed to validate implant durability, optimize design protocols, and determine cost-effectiveness compared to traditional grafting techniques.

## Conclusion

Custom 3D-printed glenoid implants represent a promising solution for addressing severe glenoid bone loss in both primary and revision rTSA. Our early results with restor3d custom baseplates demonstrate meaningful improvements in pain and function for most patients but also reveal a notable complication rate of 27.8%, including implant failures. These complications were associated with fewer and shorter large-diameter screws, underscoring the critical role of fixation strategy in implant success. While custom implants provide the advantage of immediate mechanical stability and eliminate the need for structural graft incorporation, long-term durability remains dependent on reliable osseointegration at the implant–bone interface. Future research should aim to optimize design parameters and confirm durability with larger, longer-term studies.

## Disclaimers:

Funding: Post-operative CT scans were funded by Restor3D, Inc. (paid directly to Northwest Radiology).

Conflicts of interest: Benjamin Wesorick serves as a consultant and holds stock options in Restor3D; no financial payments were received related to this article. Brian Badman serves as a consultant, lecturer, and receives royalties for Enovis, Atreon, and Tigon; receives grant support from Zimmer Biomet, Anika, and Lima; and holds stock options in Restor3D. Any additional authors, their immediate families, and any research foundations with which they are affiliated have not received any financial payments or other benefits from any commercial entity related to the subject of this article.
